# (*E*)-3-Phenyl-2-(1-tosyl-1*H*-indol-3-ylcarbon­yl)acrylonitrile

**DOI:** 10.1107/S1600536812004886

**Published:** 2012-02-10

**Authors:** S. Paramasivam, G. Bhaskar, P. R. Seshadri, P. T. Perumal

**Affiliations:** a Postgraduate and Research Department of Physics, Agurchand Manmull Jain College, Chennai 600 114, India; b Organic Chemistry Division, Central Leather Research Institute, Chennai 600 020, India

## Abstract

In the title compound, C_25_H_18_N_2_O_3_S, the indole moiety is planar and makes a dihedral angle of 89.95 (09)° with the phenyl ring of the sulfonyl substituent. The mol­ecular conformation features a weak C—H⋯N short contact and the crystal packing reveals a weak C—H⋯O hydrogen bond.

## Related literature

For the biological activity of indole derivatives, see: Andreani *et al.* (2001 ▶); Chai *et al.* (2006 ▶); Kolocouris *et al.* (1994 ▶); Ma *et al.* (2001 ▶); Nieto *et al.* (2005 ▶); Singh *et al.* (2000 ▶). For the bond-length difference, see: Allen (1981 ▶); Govindasamy *et al.* (1998 ▶); Sankaranarayanan *et al.* (2000 ▶). For the N atom-hybridization, see: Beddoes *et al.* (1986 ▶). For related structures, see: Seshadri *et al.* (2002 ▶).
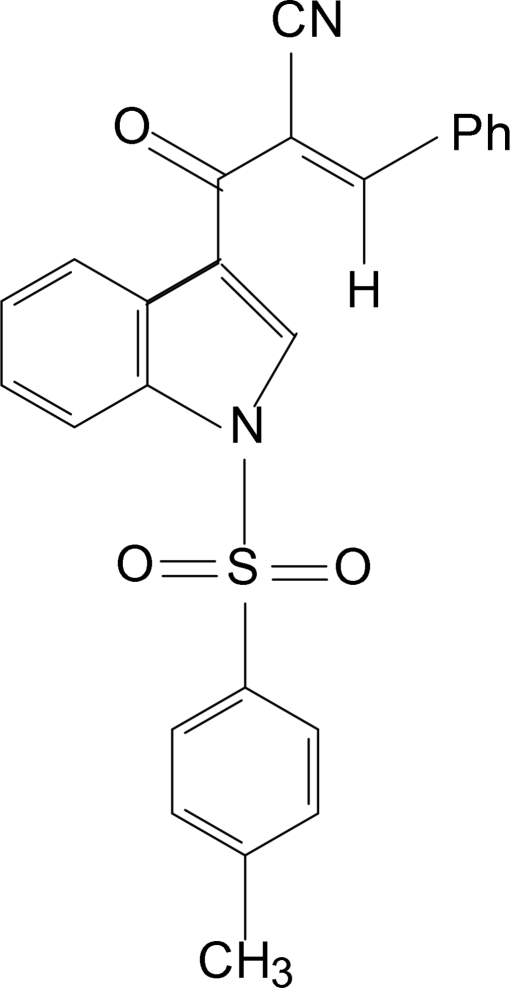


## Experimental

### 

#### Crystal data

C_25_H_18_N_2_O_3_S
*M* *_r_* = 426.47
Monoclinic, 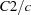

*a* = 33.8741 (13) Å
*b* = 7.1294 (3) Å
*c* = 19.9180 (9) Å
β = 118.4170 (2)°
*V* = 4230.6 (3) Å^3^
*Z* = 8
Mo *K*α radiation
μ = 0.18 mm^−1^
*T* = 298 K
0.21 × 0.19 × 0.15 mm

#### Data collection

Bruker SMART APEXII area-detector diffractometer
19948 measured reflections
5285 independent reflections
2177 reflections with *I* > 2σ(*I*)
*R*_int_ = 0.066

#### Refinement

*R*[*F* ^2^ > 2σ(*F* ^2^)] = 0.051
*wR*(*F* ^2^) = 0.176
*S* = 0.95
5285 reflections
281 parameters
H-atom parameters constrained
Δρ_max_ = 0.25 e Å^−3^
Δρ_min_ = −0.25 e Å^−3^

### 

Data collection: *APEX2* (Bruker, 2008 ▶); cell refinement: *SAINT* (Bruker, 2008 ▶); data reduction: *SAINT*; program(s) used to solve structure: *SHELXS97* (Sheldrick, 2008 ▶); program(s) used to refine structure: *SHELXL97* (Sheldrick, 2008 ▶); molecular graphics: *ORTEP-3* (Farrugia, 1997 ▶) and *PLATON* (Spek, 2009 ▶); software used to prepare material for publication: *SHELXL97*, *PLATON* and *publCIF* (Westrip, 2010 ▶).

## Supplementary Material

Crystal structure: contains datablock(s) I, global. DOI: 10.1107/S1600536812004886/kp2387sup1.cif

Structure factors: contains datablock(s) I. DOI: 10.1107/S1600536812004886/kp2387Isup2.hkl

Supplementary material file. DOI: 10.1107/S1600536812004886/kp2387Isup3.cml

Additional supplementary materials:  crystallographic information; 3D view; checkCIF report

## Figures and Tables

**Table 1:** Hydrogen-bond geometry (Å, °)

*D*—H⋯*A*	*D*—H	H⋯*A*	*D*⋯*A*	*D*—H⋯*A*
C18—H18⋯N2	0.93	2.73	3.433 (4)	133
C5—H5⋯O3^i^	0.93	2.86	3.551 (4)	132

Symmetry code: (i) 
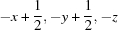
.

**Fig. 1.**
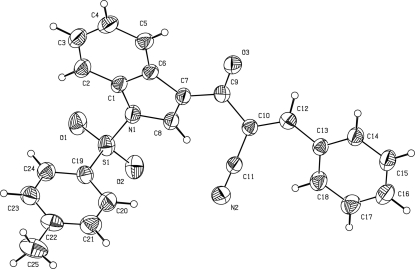
The molecular structure of the title compound, showing the atom-numbering scheme and displacement ellipsoids drawn at the 30% probability level.

## supplementary crystallographic information

### Comment

Indole derivatives exhibit anti-bacterial (Nieto *et al.*, 2005), anti-cancer, anti-malarial and anti-hypertensive (Ma *et al.*, 2001) activities. In addition, indole derivatives are known to exhibit anti-fungal (Singh *et al.*, 2000), anti-tumour (Andreani *et al.*, 2001), anti-viral (Kolocouris *et al.*, 1994) and anti-hepatitis B virus (Chai *et al.*, 2006) activities. Against this background, the title compound was chosen for X-ray structure analysis (Fig.1).

The indole ring is planar and the sulfonyl bound phenyl ring is perpendicular to the nine membered indole moiety with a dihedral angle of 89.95 (09)°.

The torsion angles O1—S1—N1—C1 and O2—S1—N1—C7 [45.9 (3)° and -23.3 (3)°, respectively] indicates the syn conformation of the sulfonyl moiety. The sum of the bond angles around N1 [357.95 (23)°] indicates sp^2^ hybridization (Beddoes *et al.*,1986).

A distorted tetrahedral geometry [O1—S1—O2 = 120.75 (13)° and O1—S1—N1 = 104.53 (12)°] around S1 is observed. The widening of the angles may be due to repulsive interactions between the two short S═O bonds.

In the benzene ring of the indole system, the *endo*-cyclic angles at C2, C5 and C6 are contracted to 116.9 (3)°, 118.6 (3)° and 118.5 (3)° respectively, while those at C1, C3 and C4 expanded to 123.3 (3)°, 121.5 (3)° and 121.2 (3)° respectively. This may be due to a real effect caused by the fusion of the smaller pyrrole ring to the six membered benzene ring, and the strain is taken up by angular distortion rather than by bond length distortions (Allen, 1981). A similar effect has also been observed by Sankaranarayanan *et al.* (2000) and Seshadri *et al.* (2002).

The difference in C—N bond lengths may be due to the electron-withdrawing character of the phenyl sulfonyl group (Govindasamy *et al.*, 1998; Seshadri *et al.*, 2002). The molecular structure is stabilised by a weak C—H···N intramolecular interactions and the crystal packing reveals a weak C—H···O hydrogen bond.

### Experimental

To the mixture of cyanoacetylindole(2 mmol) and benzaldehyde (2.1 mmol) sodium methoxide (10 mol %) was added in methanol. The mixture was allowed to reflux for 2 h. After completion of the reaction, which was washed with water and extracted with ethylacetate (10 × 2 = 20 ml), the filtrate was dried with sodium sulfate and concentrated. The crude was subjected to column chromatography to obtain pure condensed chalcone product. To the chalcone (1 mmol) which was suspended in 10 ml benzene add aqueous 30% NaOH (10 ml), containing tosyl chloride (1.1 mmol) and tetrabutylammonium bromide (0.10 mmol). After stirring vigorously for 30 min, the layers were separated and the water layer was extracted with benzene (10 ml). The combined organic layers were dried over Na_2_SO_4_ and the solvent was removed under reduced pressure. The crude product was purified by recrystallisation from CH_2_Cl_2_/hexane to afford (*E*)-3-phenyl-2-(1-tosyl-1*H*-indole-3-carbonyl)acrylonitrile.

### Refinement

H atoms were positioned geometrically and allowed to ride on their parent atoms, with C—H = 0.93–0.97 Å and *U*_iso_(H) = 1.5*U*_eq_(C) for methyl H atoms and 1.2 *U*_eq_(C) for other H atoms.

### Crystal data

**Table:**

C_25_H_18_N_2_O_3_S	*F*(000) = 1776
*M**_r_* = 426.47	*D*_x_ = 1.339 Mg m^−^^3^
Monoclinic, *C*2/*c*	Mo *K*α radiation, λ = 0.71073 Å
Hall symbol: -C 2yc	Cell parameters from 5285 reflections
*a* = 33.8741 (13) Å	θ = 1.4–28.4°
*b* = 7.1294 (3) Å	µ = 0.18 mm^−^^1^
*c* = 19.9180 (9) Å	*T* = 298 K
β = 118.4170 (2)°	Block, colourless
*V* = 4230.6 (3) Å^3^	0.21 × 0.19 × 0.15 mm
*Z* = 8	

### Data collection

**Table:**

Bruker SMART APEXII area-detector diffractometer	2177 reflections with *I* > 2σ(*I*)
Radiation source: fine-focus sealed tube	*R*_int_ = 0.066
Graphite monochromator	θ_max_ = 28.4°, θ_min_ = 1.4°
ω and φ scans	*h* = −44→45
19948 measured reflections	*k* = −9→9
5285 independent reflections	*l* = −26→21

### Refinement

**Table:**

Refinement on *F*^2^	Primary atom site location: structure-invariant direct methods
Least-squares matrix: full	Secondary atom site location: difference Fourier map
*R*[*F*^2^ > 2σ(*F*^2^)] = 0.051	Hydrogen site location: inferred from neighbouring sites
*wR*(*F*^2^) = 0.176	H-atom parameters constrained
*S* = 0.95	*w* = 1/[σ^2^(*F*_o_^2^) + (0.0805*P*)^2^] where *P* = (*F*_o_^2^ + 2*F*_c_^2^)/3
5285 reflections	(Δ/σ)_max_ < 0.001
281 parameters	Δρ_max_ = 0.25 e Å^−^^3^
0 restraints	Δρ_min_ = −0.25 e Å^−^^3^

### Special details

**Table:**

Geometry. All e.s.d.'s (except the e.s.d. in the dihedral angle between two l.s. planes) are estimated using the full covariance matrix. The cell e.s.d.'s are taken into account individually in the estimation of e.s.d.'s in distances, angles and torsion angles; correlations between e.s.d.'s in cell parameters are only used when they are defined by crystal symmetry. An approximate (isotropic) treatment of cell e.s.d.'s is used for estimating e.s.d.'s involving l.s. planes.
Refinement. Refinement of *F*^2^ against ALL reflections. The weighted *R*-factor *wR* and goodness of fit *S* are based on *F*^2^, conventional *R*-factors *R* are based on *F*, with *F* set to zero for negative *F*^2^. The threshold expression of *F*^2^ > σ(*F*^2^) is used only for calculating *R*-factors(gt) *etc*. and is not relevant to the choice of reflections for refinement. *R*-factors based on *F*^2^ are statistically about twice as large as those based on *F*, and *R*-factors based on ALL data will be even larger.

### Fractional atomic coordinates and isotropic or equivalent isotropic displacement parameters (Å^2^)

**Table:**

	*x*	*y*	*z*	*U*_iso_*/*U*_eq_	
C1	0.31518 (8)	0.3247 (4)	0.26078 (18)	0.0600 (7)	
C2	0.30356 (10)	0.4960 (4)	0.2794 (2)	0.0746 (9)	
H2	0.3167	0.5385	0.3296	0.090*	
C3	0.27188 (11)	0.5994 (4)	0.2206 (3)	0.0848 (10)	
H3	0.2632	0.7148	0.2310	0.102*	
C4	0.25246 (10)	0.5359 (4)	0.1457 (2)	0.0800 (9)	
H4	0.2306	0.6085	0.1071	0.096*	
C5	0.26497 (9)	0.3665 (4)	0.12750 (19)	0.0699 (8)	
H5	0.2521	0.3259	0.0771	0.084*	
C6	0.29717 (8)	0.2582 (4)	0.18621 (17)	0.0564 (7)	
C7	0.31823 (8)	0.0804 (4)	0.18886 (16)	0.0555 (7)	
C8	0.34654 (9)	0.0440 (4)	0.26354 (16)	0.0592 (7)	
H8	0.3643	−0.0626	0.2817	0.071*	
C9	0.30954 (9)	−0.0416 (4)	0.12459 (16)	0.0591 (7)	
C10	0.34231 (8)	−0.1964 (4)	0.13431 (14)	0.0536 (7)	
C11	0.38775 (10)	−0.1755 (4)	0.19112 (16)	0.0576 (7)	
C12	0.32800 (9)	−0.3435 (4)	0.08643 (15)	0.0578 (7)	
H12	0.2983	−0.3363	0.0482	0.069*	
C13	0.35158 (9)	−0.5115 (4)	0.08504 (15)	0.0546 (7)	
C14	0.33458 (9)	−0.6116 (4)	0.01750 (17)	0.0658 (8)	
H14	0.3087	−0.5700	−0.0248	0.079*	
C15	0.35560 (11)	−0.7728 (4)	0.0120 (2)	0.0774 (9)	
H15	0.3440	−0.8379	−0.0339	0.093*	
C16	0.39340 (11)	−0.8362 (4)	0.0741 (2)	0.0812 (9)	
H16	0.4079	−0.9431	0.0700	0.097*	
C17	0.41011 (11)	−0.7421 (4)	0.1426 (2)	0.0809 (9)	
H17	0.4353	−0.7876	0.1852	0.097*	
C18	0.38943 (10)	−0.5804 (4)	0.14814 (17)	0.0713 (8)	
H18	0.4009	−0.5170	0.1945	0.086*	
C19	0.42361 (9)	0.3581 (4)	0.39664 (15)	0.0606 (7)	
C20	0.45161 (10)	0.2880 (5)	0.37005 (18)	0.0793 (9)	
H20	0.4490	0.1638	0.3541	0.095*	
C21	0.48304 (11)	0.4022 (6)	0.36733 (19)	0.0887 (10)	
H21	0.5017	0.3542	0.3493	0.106*	
C22	0.48793 (10)	0.5860 (5)	0.39046 (18)	0.0789 (10)	
C23	0.45972 (10)	0.6538 (5)	0.41689 (19)	0.0803 (9)	
H23	0.4625	0.7779	0.4329	0.096*	
C24	0.42765 (10)	0.5417 (4)	0.42011 (17)	0.0716 (8)	
H24	0.4089	0.5896	0.4380	0.086*	
C25	0.52268 (11)	0.7124 (6)	0.3866 (2)	0.1128 (13)	
H25A	0.5393	0.6429	0.3672	0.169*	
H25B	0.5428	0.7582	0.4368	0.169*	
H25C	0.5080	0.8164	0.3534	0.169*	
N1	0.34515 (7)	0.1880 (3)	0.30873 (13)	0.0630 (6)	
N2	0.42442 (9)	−0.1524 (3)	0.23564 (16)	0.0792 (8)	
O1	0.36234 (7)	0.3040 (3)	0.43741 (12)	0.0896 (7)	
O2	0.40187 (7)	0.0271 (3)	0.42345 (11)	0.0830 (6)	
O3	0.27557 (7)	−0.0232 (3)	0.06329 (12)	0.0832 (6)	
S1	0.38381 (3)	0.21122 (11)	0.40061 (4)	0.0698 (3)	

### Atomic displacement parameters (Å^2^)

**Table:**

	*U*^11^	*U*^22^	*U*^33^	*U*^12^	*U*^13^	*U*^23^
C1	0.0581 (16)	0.0485 (17)	0.088 (2)	−0.0027 (14)	0.0464 (16)	−0.0045 (16)
C2	0.0714 (19)	0.064 (2)	0.105 (2)	−0.0046 (17)	0.0555 (19)	−0.0165 (19)
C3	0.079 (2)	0.058 (2)	0.142 (3)	−0.0015 (18)	0.072 (2)	−0.009 (2)
C4	0.0670 (19)	0.060 (2)	0.120 (3)	0.0096 (16)	0.051 (2)	0.013 (2)
C5	0.0605 (17)	0.0593 (19)	0.096 (2)	0.0004 (15)	0.0419 (17)	0.0019 (17)
C6	0.0508 (15)	0.0480 (16)	0.077 (2)	−0.0012 (13)	0.0361 (15)	0.0000 (15)
C7	0.0586 (16)	0.0462 (16)	0.0676 (18)	−0.0012 (13)	0.0347 (15)	−0.0047 (14)
C8	0.0658 (17)	0.0487 (16)	0.0690 (19)	0.0006 (13)	0.0370 (16)	−0.0050 (14)
C9	0.0588 (16)	0.0520 (17)	0.0646 (18)	−0.0011 (14)	0.0277 (15)	−0.0014 (14)
C10	0.0556 (15)	0.0493 (16)	0.0567 (16)	0.0007 (13)	0.0275 (14)	0.0008 (13)
C11	0.0592 (18)	0.0532 (17)	0.0608 (17)	0.0045 (14)	0.0288 (16)	−0.0009 (14)
C12	0.0603 (16)	0.0562 (17)	0.0595 (17)	−0.0012 (14)	0.0305 (14)	0.0026 (14)
C13	0.0584 (16)	0.0473 (16)	0.0651 (17)	−0.0017 (13)	0.0352 (14)	−0.0012 (14)
C14	0.0714 (18)	0.0610 (18)	0.0725 (19)	−0.0006 (15)	0.0403 (16)	−0.0085 (15)
C15	0.093 (2)	0.064 (2)	0.090 (2)	−0.0005 (18)	0.055 (2)	−0.0148 (18)
C16	0.090 (2)	0.059 (2)	0.117 (3)	0.0085 (18)	0.067 (2)	0.002 (2)
C17	0.082 (2)	0.060 (2)	0.099 (3)	0.0091 (17)	0.042 (2)	0.0116 (19)
C18	0.081 (2)	0.0520 (18)	0.080 (2)	0.0044 (16)	0.0374 (18)	0.0024 (15)
C19	0.0656 (17)	0.0619 (19)	0.0548 (16)	−0.0023 (15)	0.0290 (14)	−0.0033 (14)
C20	0.079 (2)	0.082 (2)	0.087 (2)	−0.0003 (18)	0.0478 (19)	−0.0138 (18)
C21	0.075 (2)	0.110 (3)	0.093 (2)	−0.002 (2)	0.050 (2)	−0.007 (2)
C22	0.0578 (18)	0.095 (3)	0.070 (2)	−0.0026 (18)	0.0193 (16)	0.0178 (19)
C23	0.072 (2)	0.065 (2)	0.089 (2)	−0.0021 (17)	0.0269 (18)	0.0049 (17)
C24	0.0721 (19)	0.067 (2)	0.080 (2)	−0.0013 (16)	0.0406 (17)	−0.0026 (17)
C25	0.073 (2)	0.143 (4)	0.111 (3)	−0.020 (2)	0.034 (2)	0.029 (3)
N1	0.0689 (14)	0.0563 (14)	0.0699 (15)	−0.0030 (12)	0.0381 (13)	−0.0106 (13)
N2	0.0704 (17)	0.0706 (17)	0.0851 (18)	0.0043 (14)	0.0276 (15)	0.0005 (14)
O1	0.1087 (16)	0.0969 (17)	0.0961 (16)	−0.0169 (13)	0.0755 (14)	−0.0299 (13)
O2	0.1187 (17)	0.0612 (13)	0.0717 (13)	−0.0003 (12)	0.0473 (12)	0.0074 (10)
O3	0.0740 (13)	0.0767 (14)	0.0737 (13)	0.0158 (11)	0.0147 (12)	−0.0106 (11)
S1	0.0880 (6)	0.0691 (6)	0.0650 (5)	−0.0087 (4)	0.0467 (4)	−0.0094 (4)

### Geometric parameters (Å, º)

**Table:**

C1—C2	1.386 (4)	C14—H14	0.9300
C1—C6	1.393 (4)	C15—C16	1.366 (4)
C1—N1	1.404 (3)	C15—H15	0.9300
C2—C3	1.368 (5)	C16—C17	1.378 (5)
C2—H2	0.9300	C16—H16	0.9300
C3—C4	1.389 (5)	C17—C18	1.380 (4)
C3—H3	0.9300	C17—H17	0.9300
C4—C5	1.384 (4)	C18—H18	0.9300
C4—H4	0.9300	C19—C24	1.375 (4)
C5—C6	1.393 (4)	C19—C20	1.381 (4)
C5—H5	0.9300	C19—S1	1.738 (3)
C6—C7	1.443 (3)	C20—C21	1.362 (4)
C7—C8	1.357 (4)	C20—H20	0.9300
C7—C9	1.457 (4)	C21—C22	1.373 (5)
C8—N1	1.381 (3)	C21—H21	0.9300
C8—H8	0.9300	C22—C23	1.379 (4)
C9—O3	1.223 (3)	C22—C25	1.513 (4)
C9—C10	1.511 (3)	C23—C24	1.375 (4)
C10—C12	1.343 (3)	C23—H23	0.9300
C11—N2	1.144 (3)	C24—H24	0.9300
C11—N2	1.144 (3)	C25—H25A	0.9600
C12—C13	1.447 (4)	C25—H25B	0.9600
C12—H12	0.9300	C25—H25C	0.9600
C13—C14	1.383 (4)	N1—S1	1.676 (2)
C13—C18	1.390 (4)	O1—S1	1.418 (2)
C14—C15	1.383 (4)	O2—S1	1.428 (2)
			
C2—C1—C6	123.3 (3)	C14—C15—H15	120.0
C2—C1—N1	129.4 (3)	C15—C16—C17	120.1 (3)
C6—C1—N1	107.3 (2)	C15—C16—H16	120.0
C3—C2—C1	116.9 (3)	C17—C16—H16	120.0
C3—C2—H2	121.6	C16—C17—C18	120.1 (3)
C1—C2—H2	121.6	C16—C17—H17	120.0
C2—C3—C4	121.5 (3)	C18—C17—H17	120.0
C2—C3—H3	119.3	C17—C18—C13	120.5 (3)
C4—C3—H3	119.3	C17—C18—H18	119.7
C5—C4—C3	121.2 (3)	C13—C18—H18	119.7
C5—C4—H4	119.4	C24—C19—C20	120.0 (3)
C3—C4—H4	119.4	C24—C19—S1	120.8 (2)
C4—C5—C6	118.6 (3)	C20—C19—S1	119.2 (2)
C4—C5—H5	120.7	C21—C20—C19	119.4 (3)
C6—C5—H5	120.7	C21—C20—H20	120.3
C1—C6—C5	118.5 (3)	C19—C20—H20	120.3
C1—C6—C7	107.6 (2)	C20—C21—C22	122.0 (3)
C5—C6—C7	133.9 (3)	C20—C21—H21	119.0
C8—C7—C6	106.7 (2)	C22—C21—H21	119.0
C8—C7—C9	126.2 (2)	C21—C22—C23	117.9 (3)
C6—C7—C9	127.1 (3)	C21—C22—C25	121.6 (3)
C7—C8—N1	110.3 (2)	C23—C22—C25	120.5 (4)
C7—C8—H8	124.9	C24—C23—C22	121.4 (3)
N1—C8—H8	124.9	C24—C23—H23	119.3
O3—C9—C7	120.9 (2)	C22—C23—H23	119.3
O3—C9—C10	119.4 (2)	C23—C24—C19	119.4 (3)
C7—C9—C10	119.6 (2)	C23—C24—H24	120.3
C12—C10—C11	122.4 (2)	C19—C24—H24	120.3
C12—C10—C9	119.0 (2)	C22—C25—H25A	109.5
C11—C10—C9	118.5 (2)	C22—C25—H25B	109.5
N2—C11—C10	177.4 (3)	H25A—C25—H25B	109.5
N2—C11—C10	177.4 (3)	C22—C25—H25C	109.5
C10—C12—C13	130.1 (2)	H25A—C25—H25C	109.5
C10—C12—H12	115.0	H25B—C25—H25C	109.5
C13—C12—H12	115.0	C8—N1—C1	108.1 (2)
C14—C13—C18	118.4 (3)	C8—N1—S1	122.25 (19)
C14—C13—C12	117.9 (3)	C1—N1—S1	127.6 (2)
C18—C13—C12	123.7 (3)	O1—S1—O2	120.75 (13)
C15—C14—C13	120.9 (3)	O1—S1—N1	106.49 (12)
C15—C14—H14	119.5	O2—S1—N1	104.53 (12)
C13—C14—H14	119.5	O1—S1—C19	110.00 (14)
C16—C15—C14	120.0 (3)	O2—S1—C19	110.14 (13)
C16—C15—H15	120.0	N1—S1—C19	103.28 (12)
			
C6—C1—C2—C3	1.7 (4)	C14—C15—C16—C17	1.3 (5)
N1—C1—C2—C3	−177.8 (3)	C15—C16—C17—C18	−1.9 (5)
C1—C2—C3—C4	−0.3 (4)	C16—C17—C18—C13	0.3 (5)
C2—C3—C4—C5	−1.1 (5)	C14—C13—C18—C17	1.7 (4)
C3—C4—C5—C6	1.0 (4)	C12—C13—C18—C17	179.8 (2)
C2—C1—C6—C5	−1.8 (4)	S1—C19—C20—C21	−179.6 (2)
N1—C1—C6—C5	177.8 (2)	C19—C20—C21—C22	0.1 (5)
C2—C1—C6—C7	177.9 (2)	C20—C21—C22—C23	−0.1 (5)
N1—C1—C6—C7	−2.5 (3)	C20—C21—C22—C25	−179.5 (3)
C4—C5—C6—C1	0.4 (4)	C25—C22—C23—C24	179.3 (3)
C4—C5—C6—C7	−179.3 (3)	C22—C23—C24—C19	0.1 (5)
C1—C6—C7—C8	1.8 (3)	C20—C19—C24—C23	−0.1 (4)
C5—C6—C7—C8	−178.5 (3)	S1—C19—C24—C23	179.4 (2)
C1—C6—C7—C9	179.2 (2)	C7—C8—N1—C1	−1.1 (3)
C5—C6—C7—C9	−1.1 (5)	C7—C8—N1—S1	−166.10 (19)
C6—C7—C8—N1	−0.5 (3)	C2—C1—N1—C8	−178.2 (3)
C9—C7—C8—N1	−177.9 (2)	C6—C1—N1—C8	2.2 (3)
C8—C7—C9—O3	159.9 (3)	C2—C1—N1—S1	−14.2 (4)
C6—C7—C9—O3	−17.1 (4)	C6—C1—N1—S1	166.19 (18)
C8—C7—C9—C10	−18.5 (4)	C8—N1—S1—O1	−152.2 (2)
C6—C7—C9—C10	164.5 (2)	C1—N1—S1—O1	45.9 (2)
O3—C9—C10—C12	−20.6 (4)	C8—N1—S1—O2	−23.3 (2)
C7—C9—C10—C12	157.8 (2)	C1—N1—S1—O2	174.8 (2)
O3—C9—C10—C11	155.9 (3)	C8—N1—S1—C19	91.9 (2)
C7—C9—C10—C11	−25.7 (3)	C1—N1—S1—C19	−70.0 (2)
C11—C10—C12—C13	6.3 (4)	C24—C19—S1—O1	−6.2 (3)
C9—C10—C12—C13	−177.3 (2)	C20—C19—S1—O1	173.4 (2)
C10—C12—C13—C14	−159.8 (3)	C24—C19—S1—O2	−141.6 (2)
C10—C12—C13—C18	22.1 (4)	C20—C19—S1—O2	37.9 (3)
C18—C13—C14—C15	−2.2 (4)	C24—C19—S1—N1	107.2 (2)
C12—C13—C14—C15	179.6 (2)	C20—C19—S1—N1	−73.3 (2)
C13—C14—C15—C16	0.7 (4)		

### Hydrogen-bond geometry (Å, º)

**Table:**

*D*—H···*A*	*D*—H	H···*A*	*D*···*A*	*D*—H···*A*
C18—H18···N2	0.93	2.73	3.433 (4)	133
C5—H5···O3^i^	0.93	2.86	3.551 (4)	132

Symmetry code: (i) −*x*+1/2, −*y*+1/2, −*z*.
